# Interaction between Alcohol Consumption and CYP 2C19 Gene Polymorphism in Relation to Oesophageal Squamous Cell Carcinoma

**DOI:** 10.1371/journal.pone.0043412

**Published:** 2012-09-11

**Authors:** Yun Shi, Guo-jun Luo, Li Zhang, Ji Shi, Dao-quan Zhang, Jian-min Chen, Xiao-bo Chen, Zhuo-dong Li, Qing Zhao

**Affiliations:** 1 Department of Chest Surgery; Kunming General Hospital of Chengdu Military Region, Kunming, Yunnan Province, China; 2 Department of Chest Surgery, The Sixtieth Central Hospital of the People's Liberation Army, Dali, Yunnan Province, China; 3 Department of Chest Surgery, The Fifty-ninth Central Hospital of the People's Liberation Army, Kaiyuan, Yunnan Province, China; IPO, Inst Port Oncology, Portugal

## Abstract

**Objectives:**

The purpose of this study is to explore the relationship between the interactions of CYP2C19 gene polymorphisms and several environmental factors and oesophageal squamous cell carcinoma (OSCC).

**Methods:**

In a case-control study of OSCC patients (n = 350) and healthy controls (n = 350), we investigated the roles of polymorphism in the CYP2C19 gene by the use of polymerase chain reaction - restriction fragment length polymorphism (PCR – RFLP) analysis.

**Results:**

The CYP2C19^*^3 AG+AA genotype was significantly more prevalent in OSCC patients (10.0% versus 3.43%; *P*<0.01). Multiple logistic regression analysis showed drinking (OR: 5.603, 95% CI: 3.431–11.112; *P* = 0.005) and smoking (OR: 4.341, 95% CI: 3.425–10.241; *P* = 0.001) was the independent risk factor of OSCC respectively, and there were significant interaction between CYP2C19^*^3 and drinking (OR: 8.747, 95% CI: 6.321–18.122; *P* = 0.009).

**Conclusions:**

The CYP2C19^*^3 polymorphism and OSCC were synergistically and significantly associated in Chinese Han patients.

## Introduction

Oesophageal squamous cell carcinoma (OSCC) is a common cancer among people in China [1]. Cancer registries established in the 1960s showed extremely high incidence rates of OSCC in China [2]. Known causes of OSCC, most notably tobacco and alcohol consumption could not explain this high incidence [2], [3]. It is generally accepted that OSCC is a complex disease resulting from the interaction between genetic and environmental factors [4], [5]. Several environmental risk factors such as smoking, alcohol drinking, prolonged use of wood or charcoal as sources of fuel for cooking and heating (resulting in excessive smoke inhalation), have been reported to be associated with OSCC [6]. Recent data imply that the environmental risk factors may be modified by polymorphisms in the carcinogen metabolizing genes i.e. gene-environment interactions [7].

Cytochrome P450 (CYP) epoxygenases metabolize arachidonic acid to epoxyeicosatrienoic acids (EETs). Current researches suggested that some CYP isoforms, such as CYP1A [8], CYP2B [9], CYP2C [10], CYP2D [11], participate arachidonic acid metabolism. CYP2C19, one human cytochrome P450 enzyme, is not only a clinically important metabolic enzyme responsible for the metabolism of a number of therapeutic drugs but also the key enzyme of EETs synthesis and abundantly expressed in endothelial and smooth muscle cells [12], [13] Recently, there were several studies concerning the CYP2C19 polymorphism and cancer susceptibility [14]–[17]. Isomura et al. [14] investigated the role of genetic polymorphism of CYP2C19 in a total of 114 cell lines of five gastroenterological cancers and found among the 114 cell lines, biliary tract cancer was suggested to be most strongly associated with poor metabolizers of CYP2C19. Afsar and colleagues [15] also found the frequency of CYP2C19 variant is very high in breast cancer patients. Yadav et al. [16] suggested that synergism amongst the poor metabolizers of CYP2C19 interact significantly with environmental risk factors in modifying the susceptibility to squamous cell carcinoma of head and neck (HNSCC). Bozina et al. [17] have summarized the relationships between some CYPs genetic polymorphisms and cancer risks.

Recently, a common genetic variant, CYP2C19*3 (G636A) gene was reported in some studies [18]. This polymorphism, 636 G→A substitution in the exon 4 region, changes the tryptophan codon to the termination codon that leads to the protein synthesis stopping earlier and the protein incompetency for the deficiency connective zone of the hemochrome and the substrate [18]. Several studies revealed that the CYP2C19*3 allele carriers have decreased metabolic capacity for some drugs such as omeprazole, chloroguanide, and certain tricyclic antidepressants. Individuals can be divided into two groups, poor metabolizers (PMs) and extensive metabolizers (EMs), depending on the ability of 4′-hydroxylate-S-mephenytoin.

However, the role of this gene variant in OSCC has not been sufficiently investigated. Normally, Asian people had a higher incidence of CYP2C19*3, which was usually about 13–16%, but in Caucasian people it was only 1–3% as well [19]. Therefore, it is very interesting to see if the high incidence of OSCC correlates with increased frequency of the CYP2C19*3 in Chinese population variant.

In the present study, we hypothesized that those with the CYP2C19*3 may have a higher risk for OSCC. We also speculated that there should be a synergistic interaction between the effect of several environmental factors and the genetic variation for the OSCC.

## Materials and Methods

### Ethics Statement

The present study was approved by the Ethics Committee of the Kunming General Hospital of Chengdu Military Region.

### Subjects

All the patients (n = 350) were diagnosed with histologically confirmed OSCC at Kunming General Hospital of Chengdu Military Region. Controls (n = 350) were age, sex, and geographically-matched individuals to the patients but with no obvious sign of disease. Controls with prior history of cancer were excluded. The patients and controls were recruited between 2009 and 2011. And both the patients and the controls are the Chinese Han ethnic origin.

### Sample DNA extraction

Blood samples were collected with a standard venipuncture technique and EDTA-containing tubes. DNA was extracted from peripheral vein blood leukocytes using a whole blood genome extraction kit (Beijing Boiteke Corporation) according to the manufacturer's instructions. Samples were coded to protect donor identity and to allow blinding of the investigators who carried out the genotyping.

### Data Collection

A standard questionnaire was administered to cases and controls by trained medical doctors to elicit information on demographic variables, including cigarette smoking, drinking history, fruit and vegetable intake, family history of cancer, and prolonged use of wood or charcoal as sources of fuel for cooking and heating (Excessive smoke inhalation). Persons reporting regular tobacco use in the previous 6 months were considered as smoker. Persons who were ingesting alcohol in the last 6 months were considered to be alcohol dinking [20]. A family history of OSCC is defined as having an unusually high number of close relatives with OSCC. For quantification of fruit and vegetable intake, 70 grams was considered an average portion of vegetables (both fresh and cooked vegetables included) and 100 grams was considered an average portion of fruit [21]. Participants were classified as consuming ≥2 portions/day if they regularly consumed at least one portion of fruit and one portion of vegetables per day or if they consumed at least 2 portions of fruit or 2 portions of vegetables per day.

### Genotyping of CYP2C19*3 polymorphism

Genotyping was confirmed by polymerase chain reaction (PCR)-restriction fragment length polymorphism (RFLP) analysis according to the protocol described previously [22]. Briefly, the forward primer is 5′- CATCCTGGGCTGTGCT-3′; and the reverse primer is 5′- AGGGCTTTGGAGTTTAGTG-3′; annealing temperature is 52°C. The PCR product (15 µL) was incubated with BamH I (Fermentas corporation) 5U in a total volume of 25 µL overnight at 37°C, and the resulting fragments were separated on a 1.5% agarose gel. The presence of the G636A variant creates a BamH I site producing two fragments of 263, and 133 bp. To ensure the results to be verified, we used sequenced genomic DNAs as positive controls in our assays.

### Statistical analysis

To test the hypothesis that the frequency of the CYP2C19*3 in the OSCC patients (7%) is more than that in the control subjects (2%). It was estimated that a total of 700 subjects would be needed to ensure a power of 80% to detect a 5% difference between the 2 groups using a 2-tailed test, with a sampling ratio of case: control group at 1∶1, bilateral risk set at 5%. Data analysis was performed using the computer software *Statistical Package for Social Sciences-SPSS* for Windows (version 13.0, SPSS Institute, Chicago, IL). Hardy-Weinberg equilibrium was assessed by chi-square analysis. Differences in enumeration data between the cases and the controls were analyzed using the Chi-square test. Differences in distributions of genotypes and alleles between the cases and the controls were analyzed using Chi-square test. Allele frequencies distribution was analyzed by 2×2 contingency tables using a 5% level of significance. Logistic regression analysis was used to estimate the odds ratio (*OR*) and its 95% confidence interval (*CI*) as a measure of the association between polymorphism of *CYP2C19* and risk of OSCC. We performed the gene-environmental interaction analysis according to the protocol described on the website http://jxzy.smu.edu.cn/151/PrjFile/lxb/showdown.asp?soft_id=244.

## Results

### General characteristics of the participants

General characteristics of the study population are presented in Table 1. There were not significantly different between the OSCC patients and the control subjects in age and gender (both *P*>0.05). However, there were significant differences in smoking, drinking, fruit and vegetables intake, prolonged use of wood or charcoal as sources of fuel for cooking and heating, and family history of cancer between the cases and the controls (all *P*<0.05).

**Table 1:** Clinical characteristics of study participants.

	OSCC (n = 350)	Control (n = 350)	*P*
Age (years ±SD)	59.4±12.5	60.7±12.4	0.098
Male gender (n, %)	212 (60.57)	204 (57.47)	0.365
Smoking (n, %)	237 (67.71)	165 (47.14)	<0.001
Alcohol drinker (n, %)	219 (62.57)	125(35.71)	<0.001
Fruit and vegetables intake (n, %)			
≥2 portions/day	146 (41.71)	247 (70.57)	<0.001
<2 portions/day	204 (58.39)	103 (29.43)	
Family history of cancer (n, %)	135 (38.57)	75 (21.43)	<0.001
Excessive smoke inhalation (n, %)	188 (53.71)	109 (31.14)	<0.001

### Distribution of the CYP2C19*3 genotype

The genotype distribution of CYP2C19*3 did not show any significant difference from Hardy-Weinberg equilibrium (P = 0.47).The frequency of the CYP2C19*3 A allele was significantly higher in OSCC patients than in controls (5.57% versus 1.86%; *P* = 0.004). The CYP2C19^*^3 AG+AA genotype was significantly more prevalent in OSCC patients (10.00% versus 3.43%; *P*<0.001, table 2). The power calculated for the study was 0.87.

**Table 2:** Genotype distribution of CYP2C19*3 polymorphism.

groups	n	Alelle (n, %)		Genotype (n, %)		
		A	G	AA	AG	GG
Cases	350	39 (5.57)	661 (94.43)	4 (1.14)	31 (8.86)	315 (90.00)
Controls	350	13 (1.86)	687 (98.14)	1 (0.29)	11 (3.14)	338 (96.57)

### Associations of traditional risk factors with OSCC

By univariate analysis, all these environmental factors including smoking, drinking, <2 portions/day of fruit and vegetables intake, and excessive smoke inhalation were associated with OSCC, and there were significant interactions between CYP2C19*3 polymorphism and these environmental factors(all P<0.05, table 3). However, in a multivariate analysis, we only found the interaction between CYP2C19*3 and drinking remains significant (OR: 8.747; 95%CI: 6.321–18.122; interaction *P* = 0.009; Table 4).

**Table 3:** Association of traditional risk factors with the CYP2C19*3 genotype on OSCC.

Traditional risk factors	CYP2C19*3 genotype	Cases (n)	Controls (n)	OR	95%CI
**Smoking**					
No	GG	105	177	1*	
No	AA+AG	8	8	1.69	1.033–5.462
Yes	GG	210	161	2.20	1.543–8.134
Yes	AA+AG	27	4	11.38	6.116–23.445
**Drinking**					
No	GG	115	219	1*	
No	AA+AG	16	6	3.42	2.421–8.332
Yes	GG	200	119	5.05	3.371–10.712
Yes	AA+AG	19	6	10.25	8.121–24.243
**Fruit and vegetables intake**					
≥2 portions/day	GG	128	242	1*	
≥2 portions/day	AA+AG	8	5	3.03	1.454–6.136
<2 portions/day	GG	177	96	3.49	2.100–8.342
<2 portions/day	AA+AG	27	7	7.29	3.697–13.154
**Excessive smoke inhalation**					
No	GG	153	233	1*	
No	AA+AG	9	8	1.71	1.011–3.189
Yes	GG	162	105	2.35	1.021–6.108
Yes	AA+AG	26	4	9.90	4.112–18.243

Data are presented as number of patients. CI: confidence interval; OR: odds ratio.

* as reference.

**Table 4:** Logistic regression analysis results.

Risk factors	β	SE	Wald χ^2^	*P*	*OR*	95%CI
drinking	0.651	0.413	8.012	0.005	5.603	3.431–11.112
smoking	1.321	0.322	11.023	0.001	4.341	3.425–10.241
CYP2C19* Drinking$	0.613	0.411	6.012	0.009	8.747	6.321–18.122
Fruit and vegetables intake	1.225	0.465	3.011	0.065	5.21	2.291–8.134
Excessive smoke inhalation	0.766	0.433	3.335	0.058	6.22	4.121–10.153

$ :the interaction between drinking and CYP2C19*3 A allele.

### Limitation of this study

The present study was limited by the relatively small sample size. This may have led to weak statistical significance and wide CIs when estimating odds ratios. And in the present study, we did not perform the functional experiment which is worthy doing in the future.

## Discussion

In the present study, we found the CYP2C19*3 polymorphism was associated with OSCC in Chinese population. There was also significant synergism between this polymorphism and drinking for the OSCC. To our knowledge, this is the first study to report this gene-environment interaction between drinking and the CYP2C19*3 polymorphism on the risk of OSCC in Chinese population.

Cytochrome P450s are the main drug metabolizing enzymes in human body, and are always found to participate in the metabolism of carcinogens or procarcinogens. CYP2C19 is one of the most important cytochrome P450s and is known as a key enzyme in the in vivo metabolism of a number of related hydantoins and barbiturates, as well as in the metabolism of structurally unrelated drugs such as omeprazole, lansoprazole, progunil, mephenytoin and citalopram [23]. CYP2C19*3 is a single base pair G636 A mutation in exon 4 of CYP2C19 which results in a premature stop codon [18].

In the present study, we found participants who carried the CYP2C19*3 A allele (AA or AG genotype) have a higher risk of OSCC compared with GG genotype carriers. Among participants carrying CYP2C19*3 GG genotypes, drinking was associated with about 5.603-fold higher risk (OR = 5.603, 95% CI: 3.431–11.112). However, drinking and carrying CYP2C19*3 A allele (AA or AG genotype) would be associated with an 8.747-fold risk for OSCC (OR = 8.747, 95% CI: 6.321–18.122) when compared with non-dinking CYP2C19*3 GG genotypes. These results indicates there was a significant interaction between CYP2C19*3 and dinking on the OSCC.

In addition, we also found that for the CYP2C19*3 A allele carriers, smoking, less than 2 portions per day of fruit and vegetables intake, and prolonged use of wood or charcoal as sources of fuel for cooking and heating (resulting in excessive smoke inhalation) increase a 11.38-fold, 7.29-fold, and 9.90-fold risk for OSCC, respectively. However, by multiple logistic analysis, these interactions did not remains significant.

In conclusion, we found a significant association between the CYP2C19*3 polymorphism and OSCC in Chinese people. There is significant synergetic effect between CYP2C19*3 polymorphism and drinking on the risk factor for OSCC.
